# Infant sleep hygiene counseling (sleep trial): protocol of a randomized controlled trial

**DOI:** 10.1186/s12888-016-1016-1

**Published:** 2016-09-02

**Authors:** Ina S. Santos, Diego G. Bassani, Alicia Matijasevich, Camila S. Halal, Bianca Del-Ponte, Suélen Henriques da Cruz, Luciana Anselmi, Elaine Albernaz, Michelle Fernandes, Luciana Tovo-Rodrigues, Mariangela F. Silveira, Pedro C. Hallal

**Affiliations:** 1 Postgraduate Program in Epidemiology, Federal University of Pelotas, Rua Marechal Deodoro 1160, 3rd floor, 96220020 Pelotas, RS Brazil; 2 Centre for Global Child Health, The Hospital for Sick Children and Department of Pediatrics, University of Toronto, Toronto, Canada; 3 Department of Preventive Medicine, School of Medicine, University of Sao Paulo, São Paulo, SP Brazil; 4 Hospital da Criança Conceição – Ministry of Health, Porto Alegre, RS Brazil; 5 Department of Maternal and Child Health, Faculty of Medicine, Federal University of Pelotas, Pelotas, RS Brazil; 6 Oxford Maternal and Perinatal Health Institute International Research Fellow, Nuffield Department of Obstetrics and Gynaecology, The John Radcliffe Hospital, University of Oxford, Toronto, UK; 7 Core Clinical Fellow in Paediatrics, Department of Paediatrics, Southampton General Hospital and Southampton University, Southampton, UK

**Keywords:** Sleep, Child development, Child growth

## Abstract

**Background:**

Sleep problems in childhood have been found to be associated with memory and learning impairments, irritability, difficulties in mood modulation, attention and behavioral problems, hyperactivity and impulsivity. Short sleep duration has been found to be associated with overweight and obesity in childhood. This paper describes the protocol of a behavioral intervention planned to promote healthier sleep in infants.

**Methods:**

The study is a 1:1 parallel group single-blinded randomized controlled trial enrolling a total of 552 infants at 3 months of age. The main eligibility criterion is maternal report of the infant’s sleep lasting on average less than 15 h per 24 h (daytime and nighttime sleep). Following block randomization, trained fieldworkers conduct home visits of the intervention group mothers and provide standardized advice on general practices that promote infant’s self-regulated sleep. A booklet with the intervention content to aid the mother in implementing the intervention was developed and is given to the mothers in the intervention arm. In the two days following the home visit the intervention mothers receive daily telephone calls for intervention reinforcement and at day 3 the fieldworkers conduct a reinforcement visit to support mothers’ compliance with the intervention. The main outcome assessed is the between group difference in average nighttime self-regulated sleep duration (the maximum amount of time the child stays asleep or awake without awakening the parents), at ages 6, 12 and 24 months, evaluated by means of actigraphy, activity diary records and questionnaires. The secondary outcomes are conditional linear growth between age 3–12 and 12–24 months and neurocognitive development at ages 12 and 24 months.

**Discussion:**

The negative impact of inadequate and insufficient sleep on children’s physical and mental health are unquestionable, as well as its impact on cognitive function, academic performance and behavior, all of these being factors to which children in low- and middle-income countries are at higher risk. Behavioral interventions targeting mothers and young children that can be delivered inexpensively and not requiring specialized training can help prevent future issues by reducing the risk to which these children are exposed.

**Trial registration:**

ClinicalTrial.gov NCT02788630 registered on 14 June 2016 (retrospectively registered).

## Background

Growing evidence from life-course research, especially from birth cohort studies, supports the role of early life factors on human capital [1, 2]. One of the relatively poorly understood but widely prevalent life factors affecting human capital both during childhood and adult life is the quality and duration of sleep [3]. Sleep problems, commonly presented as nighttime awakening and difficulty falling asleep, have been found to be associated with memory and learning impairments, irritability, difficulties in mood modulation, attention and behavioral problems (including aggressive behavior), hyperactivity and impulsivity [4–8].

In addition to contributing to developmental problems, chronic sleep problems can promote metabolic and hormonal disruptions resulting in deficits in linear growth [9]. The human Growth Hormone (GH), responsible for child growth, especially linear growth, has its maximum expression approximately one hour after sleep onset [10]. When the secretion of GH is impaired due to less efficient sleep patterns (total sleep time/total time in bed) or due to reductions in sleep duration there can be an increase in the weight:height ratio from both increases in weight but also due to reductions in linear growth [11]. There is evidence that early life exposure to short sleep duration is associated with overweight and obesity in childhood [12–15] and increases in linear growth were reported by two interventions that included activities to promote sleep quality in childhood [16, 17]. Sleep quality, measured using actigraphy (percent of motionless sleep time) was also associated with greater linear growth at 6 months [11].

The objective of this paper is to describe the protocol of sleep hygiene based behavioral intervention on infant sleep during the first year of life. By targeting sleep, an essential component of infant development, this proposal intends to identify an effective intervention to promote cognitive and neurological development and to reduce growth deficit. In addition, this study is innovative since there are no previous reports of sleep interventions in middle-income countries.

## Methods

### Study design

This is a 1:1 parallel group single-blinded randomized controlled trial with children that were born in the Pelotas 2015 Birth Cohort, a population-based birth cohort conducted in Pelotas, a city in Southern Brazil that enrolled 4331 newborns. As an educational intervention the study is single-blinded with only those assessing the outcomes being not aware of the status intervention/control of the study participants.

### Rationale for the study design

Randomized controlled trials are considered the gold standard design for the investigation of causal associations between interventions and outcomes. Although associations may be investigated under observational studies, causality cannot be inferred. Since this study aims at investigating the effect of a behavioral intervention to promote healthier sleep during the first year of life, a randomized controlled trial is the ideal design to achieve this objective.

### Sample size

The following parameters were set for sample size calculation: 95 % confidence interval; double-sided statistical tests; 80 % power; 1:1 ratio of intervention to control group; average self-regulated sleep period duration in the control group at 6 months as 9.70 ± 1.98 h, [18] 10.25 ± 1.90 h at 12 months, [18], and 10.30 ± 1.26 h at 24 months; [19] and self-regulated night sleep at least 30 min longer in the intervention group at 6, 12 and 24 months when compared to the control group. The greatest sample size obtained was 251 children in each group. With an added 10 % to account for losses to follow-up and for adjustments in the analyses the final number of children per arm is 276.

### Eligibility criteria

To be eligible to the trial the child needs to be a term, singleton, belonging to the Pelotas 2015 Birth Cohort, born healthy (newborns that needed intensive care after birth and with any congenital conditions are excluded) and to sleep regularly on average less than 15 h per 24 h (daytime and nighttime sleep) at three months of age (as reported by the mother). Term deliveries are defined as those happening at gestational age 37 weeks or greater (as assessed by the date of the last menstrual period or by the clinical maturity estimate based on the Dubowitz method [20] if the former was unknown or inconsistent). The information on child sleep duration per 24 h was gathered during the 3-month follow-up interview of the cohort participants, and is based on the answers to the following questions: *“How many hours does the child sleep from 7 pm to 7 am?”* and *“How many hours does the child sleep from 7 am to 7 pm?”.* Information on the number and duration of daytime naps, number of wake episodes during the night and on how long it takes to the child to fall sleep after a nocturnal awakening was also gathered.

To avoid co-intervention, and given a possible effect of the programmed maternal physical activity during pregnancy over the infants’ sleep, newborns of mothers that participated at the Physical Activity for Mothers Enrolled in Longitudinal Analysis study (PAMELA trial) [21] are not eligible to enter this study. The PAMELA is a randomized controlled trial also nested in the Pelotas 2015 pregnancy and birth cohort planned to assess the effect of regular exercise during pregnancy on the prevention of hypertension, preeclampsia and preterm birth. Eligible pregnant women between the 16th and 20th week of gestation are allocated into control group (426 women who are advised to keep their usual daily activities) or intervention group (213 women who engage in an exercise program, three sessions a week).

Also, since the sleep intervention will recommend a series of environmental improvements to ensure a restful sleep (no screen media exposure, reduced noise and light levels around bedtime), the intervention is restricted to families living in households with at least one bedroom. Children in continued use of medicines that can alter the sleep architecture and/or lead to drowsiness, such as anti-convulsivants, are not eligible.

## Outcomes definition

### Primary outcome

The main outcome is the average duration of nighttime self-regulated sleep (the maximum amount of time the child stays asleep or awake without awakening the parents), [18] at ages 6, 12 and 24 months. This outcome will be evaluated using actigraphy and sleep activity diary records (5 consecutive days before the intervention - baseline assessment) and for 5 consecutive days at ages 6, 12 and 24 months.

### Secondary outcomes

The secondary outcomes that will be measured are linear growth between ages 3–12 and 12–24 months and neurocognitive development at ages 12 and 24 months. The effect of the intervention on linear growth will be estimated by comparing conditional linear growth between groups. Anthropometry at ages 3, 12 and 24 months will be used for comparing the groups.

The neurodevelopment assessment designed and implemented by The International Fetal and Newborn Growth Consortium for the 21^st^ Century (INTERGROWTH-21^st^ Project) [22], The Intergrowth Neurodevelopment Assessment tool (INTER-NDA) recently published [7] and the Oxford Neurodevelopment Assessment tool (OX-NDA) derived from the INTER-NDA will be used to measure cognitive, motor, language, behavioral, attention, and executive function outcomes in children at ages 12 and 24 months.

### Randomization

Randomization was performed in advance in blocks of 6 children, at the study headquarters by a person from the research team that does not have any contact with fieldworkers or with the mothers.

### Blinding

Because this is an educational intervention strict blinding of mothers, interviewers and of the fieldwork supervisor regarding the intervention or control status of mothers and children will not be possible. The study will be single-blinded, because the interviewers who will assess the outcomes will not be aware of the intervention or control status of the participants.

### The intervention

Trained visitors deliver the intervention at the mothers/caregivers households. This behavioral intervention is composed of practices that promote self-regulated sleep habits, [23, 24] including information on normal sleep behaviors during the first year of life; ideal conditions to promote sleep onset: environmental improvements that ensure restful sleep (no screen media exposure, reduced noise and light levels); calming naptime routines and avoiding stimulating or stressing children before naptime; practices that promote child self-regulation of sleep, including putting infants to sleep drowsy but awake; and advice on how to handle nighttime awakenings [25]. Before delivering the content of the intervention, the visitor fulfilled a checklist addressing maternal practices related to the child sleep. The mothers were then praised by positive practices and advised for those that needed to be modified.

A booklet with the intervention content in local language to aid the mother in implementing the intervention was created and handed to the mothers randomized to the intervention group. A translated version (English) of the booklet can be seen in Supplementary file 1. The content of the intervention is reinforced to the mother at the 6- and 12-month trial follow-up visits. Age-specific advice in regard to daytime napping at ages 6 and 12 months is delivered at those follow-up visits. To avoid information bias in regard to compliance to the intervention recommendations, the visitors deliver the messages of reinforcement only after the application by the study interviewers of the 6- and 12-month outcome assessment questionnaire.

## Instruments of data collection

### Questionnaires

Baseline data on maternal, family and newborn characteristics are part of the Pelotas 2015 Birth Cohort protocols. Electronic questionnaires are used to undertake the interviews (computer assisted interviews). At birth (perinatal evaluation), both the interview with the mother/caregiver and newborn evaluation were carried out within 24 h of delivery. A standardized, pre-coded questionnaire composed of 13 sections was applied to the mother and included the following modules: identification; delivery and newborn health; antenatal care and gestational morbidity; reproductive history; mother’s characteristics and lifestyle; characteristics of work, father, and family income; tests taken by mother during antenatal care; newborn physical examination; and contact data for future follow-up.

The first 2015 cohort follow-up (at age 3 months) started on April 1^st^, 2015. All children in the cohort were sought for this follow-up visit. A seven-day window period was defined, including the day on which the child completed three months and the three days before and after this date. The structure of the 3-month follow-up questionnaire is similar to that of the perinatal questionnaire. An additional visit at 6 months of age was made to children enrolled in this trial.

The questionnaires collect information about parental characteristics: age, education, economic status, number of rooms at home, total number of persons living at home, number of siblings living at home; pre-delivery and perinatal information: antenatal care attendance, health problems during pregnancy or delivery, type of delivery, gestational age at birth, birth weight; infant weight and length at 3 months, feeding method (exclusive breastfeeding, predominant breastfeeding, partial breastfeeding, or formula), infant health problems (e.g. allergies, breathing problems), daycare setting (with mother at home, with babysitter or nursery); methods used as a means of soothing the infant for night and day (breastfeeding, bottle feeding, rocking); bed-sharing with the mother; and quality of the child sleep as perceived by the mother.

### Brief Infant Sleep Questionnaire (BISQ) [26]

The BISQ questionnaire is a tool for screening sleep disorders in infants and toddlers (0–3 years). The instrument was developed and validated against sleep diary and actigraphic measures by Sadeh A. [27] The translation to Brazilian Portuguese and the back-translation of the BISQ questionnaire were published by Nunes ML et al. [26] The questionnaire variables include: nocturnal sleep duration (between the hours of 7 pm and 7 am); daytime sleep duration (between the hours of 7 am and 7 pm); number of night wakings; duration of wakefulness during the night hours (10 pm to 6 am); nocturnal sleep-onset time (the clock time at which the child falls asleep for the night); settling time (latency to falling asleep for the night); method of falling asleep; location of sleep; preferred body position; child age and gender; birth order; and role of responder (who completed the BISQ). Completion of the BISQ requires 5 to 10 min. The parents are instructed to report on their child’s sleep during the past week. Poor sleepers are defined as children who present one or more of the following criteria: wake more than 3 times per night; nocturnal wakefulness period greater than 1 h; or total sleep time shorter than 9 h. The BISQ questionnaire is applied to the mother at the baseline interview and at the outcome evaluation visits at ages 6, 12 and 24 months.

### Actigraphy

At baseline the children enrolled in the trial wear an actigraph (Actiwatch wGT3X-BT) in the left ankle (even while bathing) during five days to register the mean duration of nighttime sleep. The children will wear the actigraph again during five days in each of the outcome visits (at the 6, 12 and 24 months of age). Actigraphy is an objective and validated method for assessing sleep–wake patterns in infants, children and adults. The actigraph is a wristwatch-like device that records body motion data that can be translated to reliable and valid sleep–wake measures. It informs about nighttime awakenings and sleep duration; it is simple to use, without requirements for specialized professional, safe, and comfortable for the child to wear [28, 29]. The following sleep measures will be used: total night sleep period, from sleep onset time to morning awakening time; true sleep time, sleep time excluding all periods of wakefulness at night; number of night-wakings (lasting five minutes or longer).

### 5-day activity diary

In this diary mothers from intervention and control group register the times of the day the child is asleep, awake, napping, or feeding. The diaries are provided to the mothers just after their consent to participate in the trial and before the opening of the child allocation status (intervention or control group). Mothers from the two groups will complete those diaries for a 5-day period.

### 3-day activity diary

To promote and to measure the maternal adherence to the intervention, the mothers in the intervention group receive an expanded version of the 5-day diary to be completed registering the number of nighttime awakenings, duration of nighttime awakenings, and measures taken to aid the child back to sleep. Those diaries are completed during three days starting at day 5 post-consent. Only mothers from the intervention group fulfill these diaries.

### Anthropometry

At 3, 12 and 24 months of age anthropometric evaluation will include measures of weight and length. Mother and child weight are measured using an electronic scale (150 kg capacity and 100 g precision), the mother being weighed first alone then holding the baby. The child’s weight is calculated as the difference between the two measures. The mother is weighed clothed, but without heavy outfits, and clothes worn by the mother is recorded. The child is weighed undressed, whenever allowed by the mother. Otherwise, the child’s clothing is recorded.

Length is measured using a foldable wooden infantometer, custom made for the study, using for measurement a nylon tape measure with 1 mm precision adhered to a groove carved into the body of the instrument.

### The Intergrowth Neurodevelopment Assessment (INTER-NDA) and The Oxford Neurodevelopment Assessment (OX-NDA) tools

The Intergrowth Neurodevelopment Assessment tool (INTER-NDA) [7] is a measure of cognition, motor skills (fine and gross motor), language (expressive and receptive), behavior, executive function, attention, and social-emotional reactivity for children. The 24-month version of the INTER-NDA covers the 22–30 months age-range and was designed and implemented by the INTERGROWTH-21^st^ Project under which more than 1100 children in Brazil, India, Italy, Kenya, and the UK have been assessed. The INTER-NDA has been validated against the Bayley Scales of Infant and Toddler Development, Third Edition (BSID-III scales), [30] and has been evaluated for reliability, internal consistency and for administration by non-specialist field workers.

The OX-NDA is an extension of the 24-month-INTER-NDA to younger age ranges. It covers the 10–14 month age group. It is a 57-item measure consisting of a combination of directly administered, concurrently observed and maternally reported items. Both the 12 and 24 month versions of the NDA were designed to be international, population-based screening measures for early child neurodevelopment. They are based on objective reporting by (rather than the subjective judgment of) the examiner on the child’s performance and aims to characterize child outcomes across a spectrum. The child’s performance on each item is reported on a five-point scale. The INTER-NDA yields mean cognitive, language, motor, attention and social-emotional reactivity scores; as well as positive, negative and global behavior composites and sub-scale scores for receptive language, expressive language, fine motor skills and gross motor skills. Both versions of the NDA (i.e., at 12 and 24 months) are designed to be administered without the need for specialist personnel or infrastructure, with an administration time of 15–20 min. The OX-NDA will have its validity assessed in a sub-study specifically planned to this end that will be nested in the trial.

### Logistics of the study

**Fig. 1:**
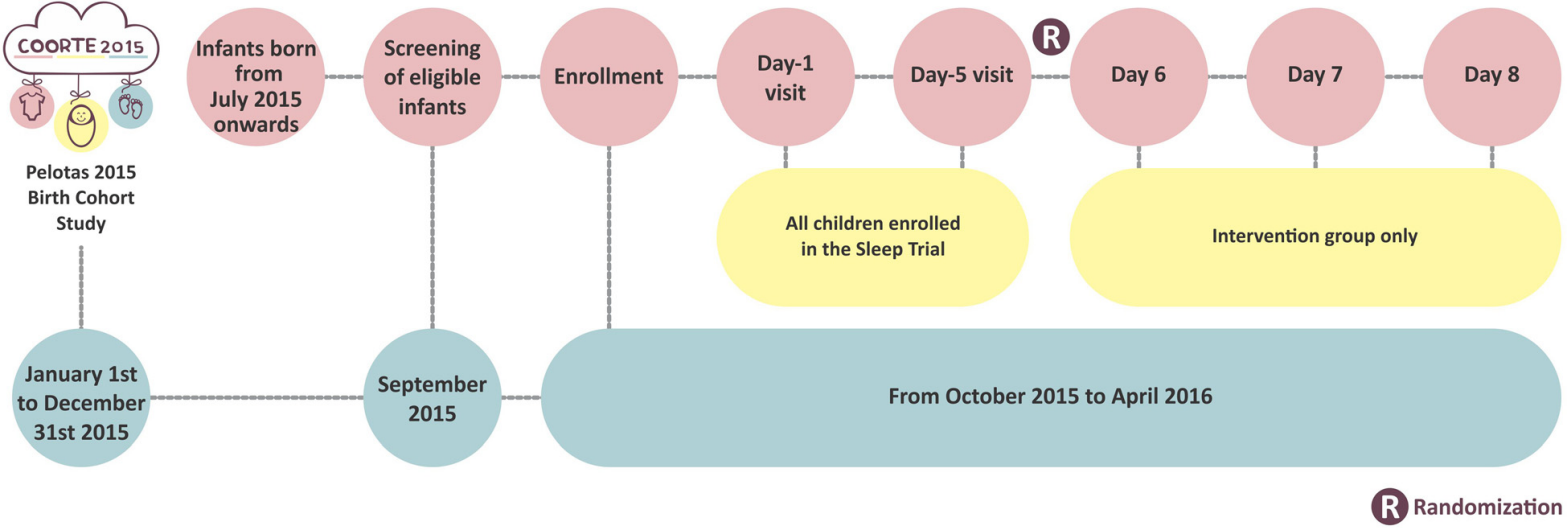
Trial flowchart of study procedures for both groups

Figure 1 shows the timeline of study procedures implementation. Enrollment to the trial started on October 2015. At the 3-month follow-up of the cohort eligible children born from July to the end of December 2015 were identified. Eligible infants are identified at the study headquarters by the study supervisors. Following identification of eligibility, the mothers of eligible infants were contacted by phone and invited to receive a visit from one of the sleep trial visitors. For those who accepted to participate, a household visit was scheduled for one the following days. A trained visitor paid the visit to the mother, explained in general terms the objective of the study and invited the mother to participate in the study. Those who agreed to participate were presented with the informed Consent Form (CF) for signature and a follow-up visit was scheduled within five days. Between CF signature and the 5-day visit, the child used the actigraph on the left ankle. The mother was oriented on how to fill out the activity diary registering the times of the day the child was asleep, awake, napping, and feeding.

For each child enrolled in the study, the visitor received an opaque envelope, sealed, that was only opened at the 5-day visit to reveal the child allocation in the trial (intervention or control group). At this visit, the actigraphs and the 5-day diaries were collected and the sleep recommendations were delivered to the mothers from the intervention group. At the 5-day visit, the mothers of infants randomly allocated to the control group received a printed educational material about maternal and child benefits associated to breastfeeding and were informed about the follow-up visits to be paid at ages 6, 12 and 24 months.

**Fig. 2:**
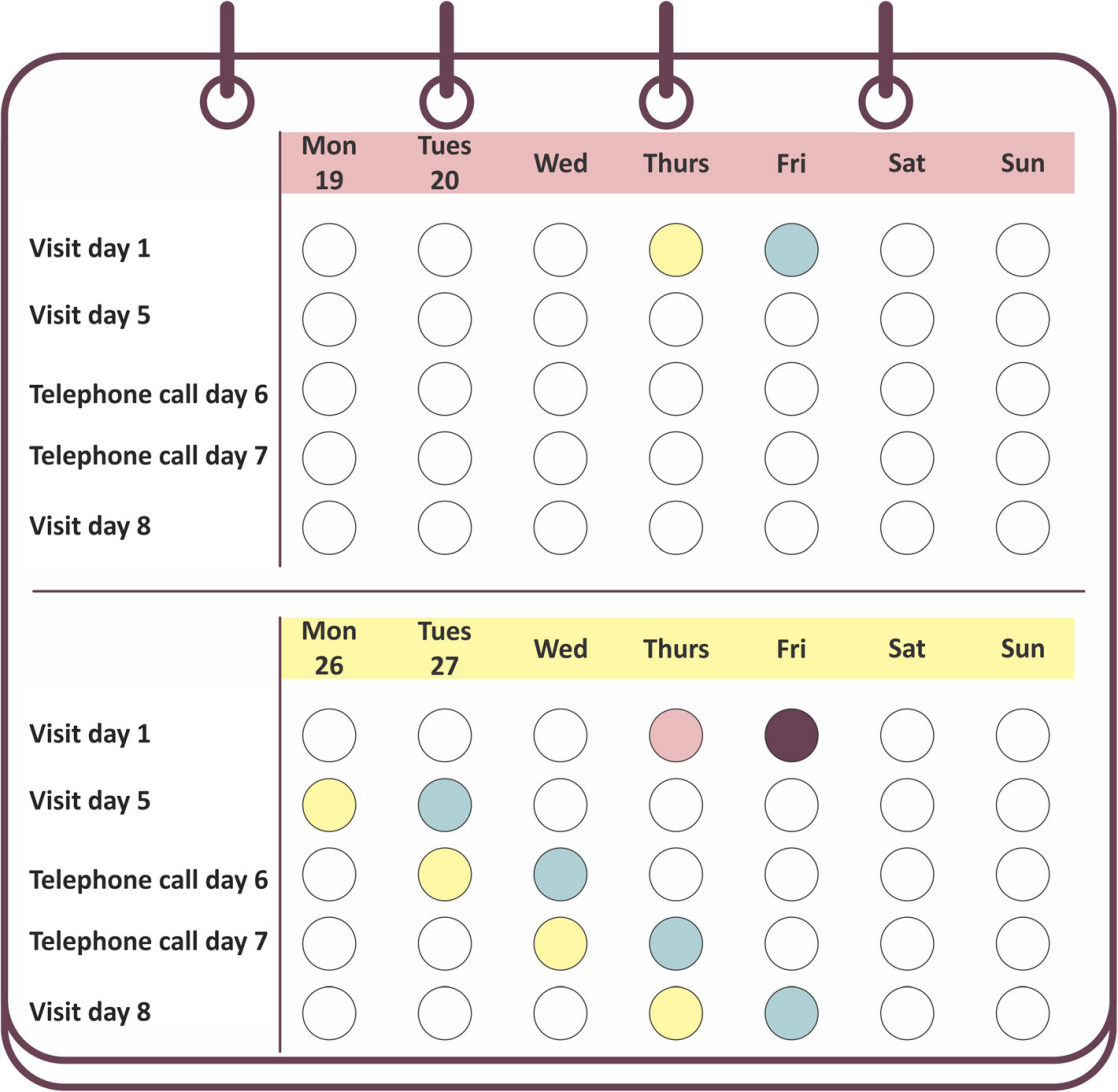
Timeline for household visits and telephone calls for mothers from the intervention group

To improve adherence to intervention protocol mothers from the intervention group received a phone call on days 1 and 2 following the delivery of the intervention to check and support for possible maternal difficulties in implementing the recommended actions and to reinforce the content of the intervention. The same visitor who delivered the intervention paid those phone calls that were made from the study headquarters. Mothers from the intervention group received a new diary to be fulfilled during the three days following the delivery of the intervention. This diary collected information on the number of nighttime awakenings, duration of nighttime awakenings, and measures taken to aid the child back to sleep. At day 8 post-CF signatures the visitor paid a new visit to the mothers from intervention group with the aim of collecting diaries, assess maternal impressions about the child’s sleep, to aid in managing difficulties implementing the intervention, and in managing the child’s sleep. At this visit the mother had the opportunity to solve any doubts about the implementation of the intervention and about the use of the diary. To support mothers from intervention group on any infant sleep related issues that arose at any time after the delivery of the intervention, a telephone number to contact the study team was provided. The timeline for household visits and telephone calls for mothers from the intervention group is shown on Fig. 2. As shown in the Fig. 2, to avoid household visits on weekend days, day-1 visits were paid only on Thursdays and Fridays.

**Fig. 3:**
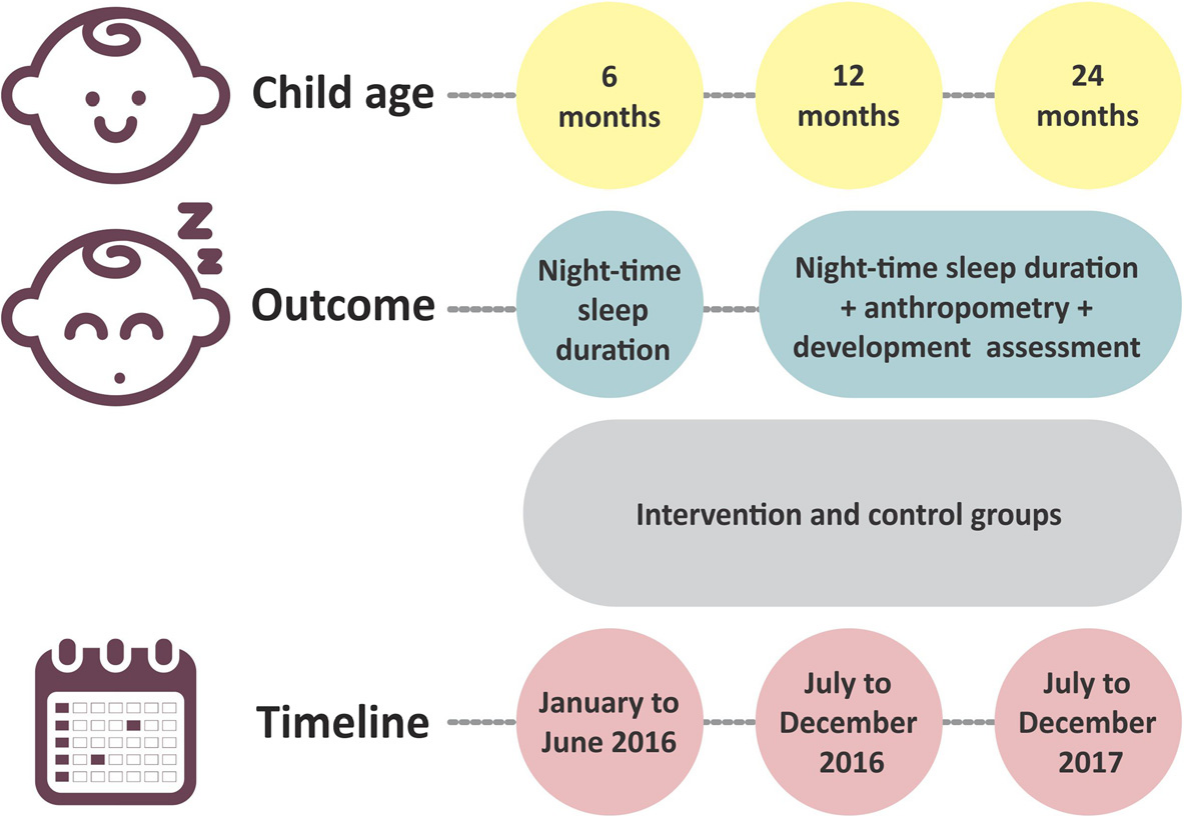
Timeline for study outcomes

At 6, 12 and 24 months of age, all study children (intervention and control group) will have measured for five days the duration of self-regulated nighttime sleep by means of actigraphy. At 12 and 24 months of age all study children (intervention and control group) will undergo anthropometry and neurodevelopment assessment. Figure 3 shows the timeline for outcome evaluations.

### Study team

The fieldwork team is composed of visitors, interviewers, fieldwork supervisors, and coordinators. All visitors were females with graduation in Nursing, Nutrition or Pedagogy. Visitors training included general instructions in regard to the study protocol, reading of the questionnaire and fieldwork manual, detailed discussion of each question, and practice questionnaire administration among themselves and with volunteer mothers (not participating in the study). Special focus was given to the training of visitors on the delivery of the intervention: written training material focusing on normal sleep behaviors, how to facilitate sleep onset and how to manage night awakenings were produced to the intervention training. Two Pediatricians, two Psychologists, one Nutritionist and one Geneticist were the Facilitators at the training sections. The training program lasted 48 h, with 75 % of the time spent in practical sessions with volunteer mothers not belonging to the Pelotas 2015 Birth Cohort. The development of counseling skills was emphasized. The performance of all trainees was formally assessed at the end of the training to document its effectiveness and to aid the coordinators in the selection of the fieldworkers. For standardization proposals, the visitors were re-trained in the content and delivery of the intervention three months after the start of the fieldwork.

All interviewers have completed university level graduation. About twice the number of interviewers necessary for each visit are included in the training program. Interviewer selection is based on a written test and performance evaluation during training. The anthropometric measures are performed by the 2015 cohort fieldworkers that received specific anthropometry techniques training for assessing children aged 3, 12 and 24 months at a daycare center, until all of them were performing measurements correctly. The maximum allowed differences for acceptable precision for length is 7 mm, for weight 50 g in neonates and 100 g in older infants.

### Quality control

Quality control measures include use of pretested, standardized data collection forms; the construction of detailed visitor and interviewer guides; careful selection and evaluation of visitors and interviewers; thorough training on interviewing and anthropometric measurements, followed by standardization sessions with assessment of intra and inter-observer variability; weekly meetings of fieldwork supervisors with the visitors and interviewers; double data entry; and range checks for data values. Additionally, at a daily basis visitors and interviewers send electronically a report of the work performed during the day to the fieldwork supervisor that gives them any needed feedback.

### Pilot study

Prior to the study start, the sleep recommendations were tested in a pilot group of 20 mothers of children at the first year of life. The aim of the pilot study was to evaluate the acceptability of the recommendations and the feasibility of the study in the cultural context of child care practices in the city of Pelotas and to undertake the appropriate adjustments if necessary.

### Methodology of analysis

The intervention and control groups will be compared in terms of baseline indicators collected at the perinatal cohort interview and at the 3-month cohort interview, including child sleep habits at 3 months of age. Any differences between the two groups that had occurred despite the randomization will be adjusted for in the analyses. Analyses will be completed on an intention-to-treat basis. The Stata Statistical Software will be used in the analyses. Trial results will be communicated to healthcare professionals and other relevant groups via scientific publications in open-access peer-reviewed medical journals. To communicate the results to the community press release to local and regional newspapers containing the relevant findings of the study will be written.

## Discussion

The proposed study has strengths and limitations. One of the strengths is its feasibility. A population-based birth cohort study initiated in the same city in 2004 showed that an average of 300 children are born each month from mothers residing in the urban area of Pelotas at the city hospitals, [31] and that the mean total sleep duration (sum of daytime and nighttime) at age 3 months was 13.34 ± 2.91 h (data not published). Therefore, it was possible to obtain the necessary number of children during the year 2015 within the ongoing birth cohort study. To enable proper follow-up within the schedule of the proposal, children born between July, 1^st^ and December, 31^st^, 2015 were eligible to the trial.

This project is focused on sleep duration. The literature, especially observational studies, often does not distinguish between time in bed and actual sleep time. However, actual sleep time is typically less than time in bed, which biases data toward higher sleep duration estimates. By contrast, intervention studies using laboratory measured sleep time typically produce shorter sleep durations. This study will employ maternal records (diaries) and actigraphy to measure and compare sleep habits between intervention and control group before and after the delivery of the intervention. Those are reliable and recommended methods to assess the effect of interventions aiming to improve child sleep [7].

Additionally, as this trial is nested within a birth cohort, it will be possible to follow the trial participants in the future and assess long-term effects of the intervention, such as attained height, academic achievement, intelligence quotient, mental health, and economic productivity in adult life.

Because this is an efficacy trial, maternal compliance is mandatory for the achievement of the study objectives. However, poor maternal adherence to the recommended practices is a potential limitation of this study. After the delivery of the recommendations, mothers from the intervention group received telephone calls in two consecutive days and one additional home visit (at day 8 after the randomization) as a strategy to promote compliance. The use of diaries to record the number of nighttime awakenings, duration of nighttime awakenings, and measures undertaken to aid the child back to sleep will help to measure maternal compliance with the study. The booklet containing key messages of the intervention will help the mother remember the recommendations. Also, a telephone number to contact the study team was provided to support mothers in the implementation of the recommendations that are part of the intervention. The main outcome (self-regulated night sleep) will be assessed by maternal report, diaries and, in order to prevent information bias, by means of an objective measure (actigraphy).

It is expected that this study will demonstrate the beneficial effect of a sleep intervention on children’s sleep behavior. This is a low cost intervention that does not require specialized professional to be delivered and that can be scaled-up to high-, middle- and low-income countries. The negative impact of inadequate and insufficient sleep on children’s physical and mental health are unquestionable, as well as its impact on cognitive function, academic performance and behavior, all of these being factors to which children in low- and middle-income countries are at higher risk [32]. Behavioral interventions targeting mothers and young children that can be delivered inexpensively and that do not require specialized training can help prevent future issues by reducing the risk to which these children are exposed.

## Abbreviations

BISQ: Brief Infant Sleep Questionnaire
CF: Consent form
GH: Growth hormone
INTER-NDA: The Intergrowth Neurodevelopment Assessment tool
OX-NDA: The Oxford Neurodevelopment Assessment tool
